# Exploring quantitative measures in metacognition of emotion

**DOI:** 10.1038/s41598-023-49709-7

**Published:** 2024-01-23

**Authors:** Hsing-Hao Lee, Gabrielle Kaili-May Liu, Yi-Chuan Chen, Su-Ling Yeh

**Affiliations:** 1 Department of Psychology, New York University, New York, NY USA; 2 Department of Psychology, National Taiwan University, Taipei, Taiwan; 3 Department of Computer Science, Yale University, New Haven, CT USA; 4 Department of Brain and Cognitive Sciences, Massachusetts Institute of Technology, Cambridge, MA USA; 5 Department of Mathematics, Massachusetts Institute of Technology, Cambridge, MA USA; 6 Department of Medicine, MacKay Medical College, Taipei, Taiwan; 7 Graduate Institute of Brain and Mind Sciences, National Taiwan University, Taipei, Taiwan; 8 Neurobiology and Cognitive Science Center, National Taiwan University, Taipei, Taiwan; 9 Center for Artificial Intelligence and Advanced Robotics, National Taiwan University, Taipei, Taiwan; 10 National Humanities Center, Research Triangle Park, NC USA

**Keywords:** Human behaviour, Emotion

## Abstract

Metacognition of emotion (meta-emotion) refers to the ability to evaluate and identify one’s emotional feelings. No previous study has defined and measured this construct through objective and quantitative procedures. We established a reliable method to measure meta-emotion. With a two-interval forced-choice procedure, participants selected which of two pictures elicited stronger positive emotion; via the Law of Comparative Judgment, their responses were used to compute individual psychological distances for the emotional responses triggered by the pictures. Then, participants were asked to judge whether a pre-exposed picture induced a stronger positive emotion than the median of that elicited by the whole picture set, followed by a confidence rating. By utilizing each individual’s psychological distance, the correctness of a participant’s emotional experience was quantified by *d*ʹ, and meta-emotion was quantified using meta-*d*ʹ, *M-ratio*, and *M-diff* as indices of metacognitive sensitivity and efficiency based on Signal-Detection Theory. Test–retest reliabilities, validated by Spearman correlation, were observed in meta-*d*ʹ, *M-ratio,* and marginally with *M-diff*, suggesting the stability of meta-emotion in the current design*.* This study unveils a validated procedure to quantify meta-emotion, extendable for assessing metacognition of other subjective feelings. Nevertheless, caution is warranted in interpretation, as the measured processes may be influenced by non-metacognitive factors.

## Introduction

Understanding our own emotions as elicited by daily events allows us to appropriately adjust and regulate mood and respond to mood swings. These adjustment abilities depend on how accurately we can introspect our emotional experiences. Metacognition of emotion, also known as *meta-emotion*, is the ability to monitor and evaluate our emotional experiences in order to reduce negative emotions while reinforcing positive ones. Meta-emotion derives from metacognition, commonly known as “cognition about cognition”; that is, the ability to monitor and evaluate one’s own cognitive processing^1–3^. When external feedback is *not* available, metacognition, serving as a subjective performance evaluator, guides individuals to master new knowledge and skills by adjusting their performance accordingly^4^. This likewise applies to our affects via meta-emotion. For example, in daily social situations, we usually receive feedback from others regarding our emotional expressions, such as when friends and family attempt to provide comfort and help resolve negative emotions. Even so, in order to interact with others properly and avoid offensive behavior, we must understand and regulate our own emotions quickly and accurately.

Previous research has extensively investigated and developed methodologies for measuring metacognition of *exteroception*^2^, such as visual perception^5^ and cognitive control^6^. However, even though there have been some studies investigating the relationship between interception and metacognition^7–11^, none have specifically aimed at quantifying meta-emotion, which was the goal of the current study. Quantifying meta-emotion can allow individuals to systematically evaluate their (or others’) ability to be aware of their emotional experiences. In other words, it can help us derive a sense of our own ability to identify emotions.

Meta-emotion can also be applied to the clinical fields. For example, meta-emotion can serve as an objective index for Alexithymia, a common symptom in patients with psychological disorders such as schizophrenia^12^. Additionally, a lack of meta-emotion can lead to difficulties in emotion regulation, as identifying emotional issues is typically the initial step toward effectively managing one’s emotions. Thus, as demonstrated in individuals with depression, who often are unaware of the emergence of their negative affectivities, those with relatively weaker meta-emotion ability tend to struggle to identify and adjust their negative emotions in a timely manner^13^.

Metacognitive sensitivity and efficiency, based on the Signal-Detection Theory (SDT) framework, were first developed to quantify metacognition of visual perception^5^. Metacognitive *sensitivity* quantifies the relationship between performance and confidence rating to provide an estimation of metacognition; metacognitive *efficiency* further regresses out the influence of performance on metacognitive sensitivity, providing an unbiased measure of metacognitive processing^3^. The strength of using metacognitive efficiency as the index lies in its insusceptibility to task performance and confidence bias, which often interfere when computing correlations between performance and confidence^2^. Metacognitive efficiency has also been used to quantify metacognition of interoception. For example, Mayeli et al.^14^ asked participants to swallow a vibrating capsule into the stomach. Participants were instructed to answer if they detected the vibration from the capsule manipulated by the experimenter. Despite being invasive, this study successfully captured metacognition of gut feelings. Another study developed an alternatively non-invasive approach to evaluate the metacognition of interoception^15^. A heart rate discrimination task in which heartbeats were recorded and immediately re-played auditorily. Participants were asked to determine whether the beating rate they heard was faster or slower than their own heartbeats and how confident they were in the judgment. Legrand et al.^15^ demonstrated that metacognitive measures of heartbeat perception have high test–retest reliability and can be quantified through the SDT framework. Since these works successfully captured metacognition of interoception in the heartbeat and gut feeling domains, similar methods can be applied to work regarding meta-emotions.

We aimed to close the gap in quantifying one’s own meta-emotion using an objective, quantitative procedure and standard operationalization based on previous exteroception and interoception research. For example, in one study, participants were instructed to make a motor response to the motion direction of visual dots (e.g., “tracking the average moving direction of the random dots”), which was followed by an evaluation of their performance (e.g., “whether the performance now was better than the average of the previous block?”) and a confidence rating in the judgment^4,16^. Because performances for both the visuomotor task (type-I performance) and its evaluation (type-II performance) can be captured by the SDT framework, metacognition of exteroception performance can be quantified. In this computation, the reference or ground truth of the response is an important foundation for behavioral correctness. In studies of exteroceptions such as the above example, experimenters can easily manipulate visual motion direction and ask participants to make a perceptual judgment followed by a confidence rating. Emotional experience, by contrast, is subjective and cannot be captured without a subjective report^17^. Therefore, establishing a method to access the reference (i.e., the ground truth) for emotional judgment is a prerequisite to quantifying meta-emotion.

The ground truth of one’s subjective emotional feelings must be obtained before proceeding with the main task of assessing one’s own emotions and meta-emotion. If a physical index is not available, the Law of Comparative Judgment (LCJ) proposed by Thurstone^18^ provides an excellent psychophysical tool for quantifying human preference^19^. Individuals can express their subjective preferences in pairwise comparisons of sets of objects, and these preferences are then mathematically transformed into a continuous interval scale (i.e., the psychological distance). Thus, we used the LCJ to quantify a participant’s emotion by capturing how emotional experiences were distributed in each individual’s mind. It is worth noting that participants did not know where the ground truth was and instead had to sense it through introspection.

## Methods

### Participants

Thirty-three participants (18 males, 18–29 years old) were recruited in the current study. To achieve a robust simulation of metacognitive sensitivity and efficiency, a sample size greater than 20 is sufficient^3^. We recruited about 1.5 times as many participants as this in order to increase the power. All participants reported normal or corrected-to-normal vision, and none had histories of psychological or neurological disorders. Data from one participant was excluded from the analysis because he did not complete the task as instructed, so data from 32 participants were analyzed. Before the experiment began, participants signed an informed consent form. This study was approved by the Research Ethics Committee at National Taiwan University (NTUREC 201801HS015).

### Stimuli and apparatus

The affective stimuli were drawn from the International Affective Picture System (IAPS)^20^, which contains a collection of normative emotional pictures for valence and arousal rated on a 9-point Likert scale (valence: 1 for very negative and 9 for very positive; arousal: 1 for very calm and 9 for very arousing). Forty-five pictures were chosen from valance ratings between 6 and 7 (i.e., mildly positive) and arousal ratings between 4 and 4.5. We used this constrained range of valence and arousal ratings to generate the proper task difficulty.

Another 23 participants who did not participate in the main experiment were asked to rate the valence and arousal of these pictures on a 9-point Likert scale; this was necessary to ensure that the chosen pictures were within the expected valence and arousal ranges. We excluded pictures with mean valence ratings from this group greater than 7 or lower than 6, as well as mean arousal ratings less than 4. We ultimately obtained 20 pictures for the main experiment and four more for the practice session stage of the procedure. The mean valence and arousal ratings of the 20 pictures were 6.15 (SD = 0.43) and 4.52 (SD = 0.6), respectively. At a distance of 57 cm, the pictures were displayed in the center of the monitor on a black background (23° wide and 13° tall). Participants were instructed to rest their heads on a chin-rest during the experiment.

### Questionnaires

Three questionnaires concerning emotion expression and regulation were administered to the participants: (1) *The Alexithymia scale*, consisting of 20 items on a scale of 1 (strongly disagree) to 5 (strongly agree), was used to determine whether participants reported difficulty recognizing and describing their own emotions^21^. A higher score indicates greater difficulties in emotional expression. (2) *The positive and negative affective scale (PANAS)* was used to measure each participant’s current affective state^22^. PANAS consists of 20 descriptions, half of which are associated with positive affectivity and the other half with negative affectivity. Participants rated their current affective state on a scale of 1 (not at all) to 5 (strongly agree). A higher score in positive (negative) affectivity indicates that an individual’s emotion is more positive (negative) at test time. (3) *The emotion regulation questionnaire (ERQ)*, which consists of 10 items on a scale of 1 (strongly disagree) to 6 (strongly agree), was used to assess the ability to reappraise or suppress affects^23^. A higher score indicates better emotion regulation ability.

As the original goal of the current study included investigating the effect of meditation on emotion and meta-emotion, three additional questionnaires were administered to the participants. These were: (1) the five-facet mindfulness questionnaire (FFMQ)^24^, (2) the body awareness scale (BAS)^25^, and (3) the Oxford Happiness Questionnaire (OHQ)^26^. Due to the COVID-19 pandemic, nearly one-third of participants were unable to complete meditation training and Test 2 (see “Procedure” section) on time, so we focused on the reliability of meta-emotion measures instead.

We predicted the following correlations between emotion-oriented questionnaires and task performance. First, sensitivity to emotional experience (i.e., the *d*ʹ) was expected to be negatively correlated with the Alexithymia scale score. Further, the ability to monitor emotion (i.e., the meta-emotion) was expected to be positively correlated with the ERQ score. Finally, we considered PANAS to explore the potential relationship between current emotional state and the ability to identify and monitor emotional experience.

### Procedure

The current study was conducted as a part of a metacognition and meditation project, and each participant went through three sessions: Test 1, meditation training, and Test 2. Both Test 1 and Test 2 included the meta-emotion experiment described here, as well as a tactile experiment described in another study. The meditation training was a 5-day open-monitoring meditation course. All participants completed the three sessions within 3 weeks, aside from nine participants whose Test 2 was delayed by 3 months due to the COVID-19 pandemic. Note that Test 1 and Test 2 had the same procedure (i.e., the task and the questionnaires administered were identical).

**Figure 1:**
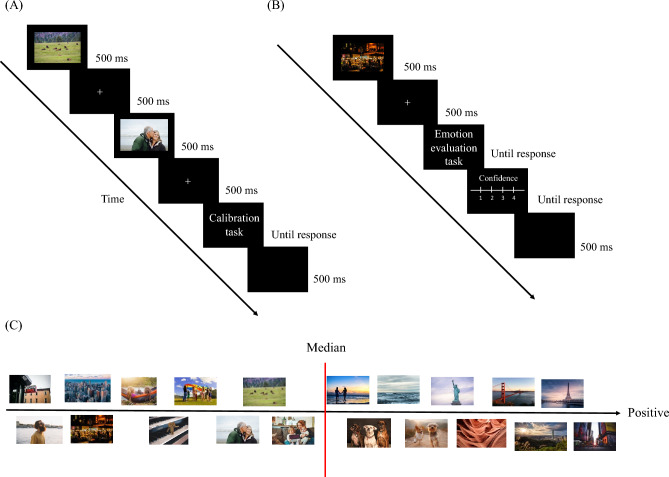
(**A**) The procedure for the calibration task. Participants were asked whether the first or the second picture elicited a stronger positive emotion. (**B**) The procedure for the emotion evaluation task. Participants were asked whether the presented picture induced a higher or lower positive emotion compared to the median of the whole picture set, followed by a confidence rating on a 4-point scale. (**C**) An example of the psychological distance map for 20 affective pictures. The pictures’ locations represent the level of positive emotion that they induced in a participant’s mind, which was quantified by the law of comparative judgment based on the responses in the calibration task. Note that the pictures presented here were not the original ones used in the current study due to IAPS copyright limitations.

The meta-emotion experiment included two tasks: calibration and emotion evaluation (see Fig. 1 for the experimental procedure). Participants completed the Alexithymia scale, PANAS, and ERQ after signing the informed consent form outside the experimental room. They then entered a sound-attenuated experimental room to perform the calibration task. The main goal of the calibration task was to quantify the psychological distance between the affective pictures individually. The psychological distance quantified the positive emotion elicited in each participant’s mind by each affective picture. Participants viewed two affective pictures sequentially for 500-ms each, separated by a 500-ms fixation. They then determined whether the first or the second picture elicited a stronger positive emotion (i.e., a two-interval forced-choice task). Following the response, the next trial started after a 500-ms inter-trial interval (ITI). Each pair combination of the 20 pictures (190 trials) was presented twice in the calibration task, yielding 380 trials. Prior to the calibration task, participants completed a practice block consisting of 12 trials in which all possible combinations of four pictures (i.e., 6 pairs) were each presented twice.

After finishing the calibration task, participants were invited to another room to complete three questionnaires (FFMQ, BAS, and OHQ, see “Questionnaires” section). This step was intended to wash away participants’ memories of their responses in the calibration task, whereby memories can be extinguished by moving to another room(s) and engaging in other tasks^27^.

Participants were then invited back to the experimental room to re-view all 20 affective pictures used in the calibration task (500 ms each, randomly presented), in order to feel the variation in positive emotion induced by individual pictures. In the subsequent emotion evaluation task (Fig. 1B), participants were presented with one affective picture from the picture set for 500 ms, followed by a 500-ms fixation and two tasks: the first was a two-alternative forced-choice (2-AFC) task regarding whether this particular picture induced a higher or lower positive emotion compared to the median of the whole picture set (type-I task); the second task was a confidence rating regarding their decision in the preceding 2-AFC task using a 4-point scale (type-II task). The scale labels were: (1) very unconfident, (2) unconfident, (3) confident, and (4) very confident. Participants were encouraged to use the full range of the confidence scale. The next trial began after a 500-ms ITI. Each picture was randomly presented three times, yielding 60 trials in total. Because the structure of the emotion evaluation task differed from that of the calibration task, it is unlikely that memory of the judgments in the calibration task would influence the result of the emotion evaluation task.

### Data analysis

#### Psychological distances for affective pictures

The responses in the calibration task were used to construct for each participant a psychological distance map of the 20 affective pictures, followed by generation of the boundary (i.e., the median) separating high versus low positive emotion inducers (10 pictures in each category) for the emotion evaluation task. Figure 1C shows an example of the psychological distance map. The psychological distance was calculated based on the LCJ^18^. Each of the 20 affective pictures was paired with one of the other 19 affective pictures, yielding a 20-by-20 frequency matrix of affective pictures for each participant that demonstrated how many times one picture outperformed another picture in inducing stronger positive emotion (Eq. (1), picture A to picture T, 20 items).

The frequency matrix was divided by the trial number and *z*-transformed to derive the psychological distance. In particular, given Eq. (2) which describes the relationship between *z*-score and psychological distance, the difference in the psychological distance for each pair of pictures (e.g., ${\overline{\psi }}_{x}-{\overline{\psi }}_{y}$) can be obtained using Eq. (3). Assuming that the psychological distance is normally distributed (${\sigma }_{{\overline{\psi }}_{x}}={\sigma }_{{\overline{\psi }}_{y}}=1$) and independent (${r}_{{\overline{\psi }}_{x}{\overline{\psi }}_{y}}=0$), Eq. (3) can be rewritten as Eq. (4). Matrix multiplication as shown in Eq. (5) can then be used to derive precise psychological distance values ($\overline{\psi }$) minus some constant *c*. This constant is the sum of all psychological distances. Given that we are concerned only with the relative psychological distances between picture-induced emotions, the constant *c* does not influence our interpretations.

1 $$\left[\begin{array}{ccccc}{f}_{AA}& {f}_{AB}& {f}_{AC}& \cdots & {f}_{AT}\\ {f}_{BA}& {f}_{BB}& {f}_{BC}& \cdots & {f}_{BT}\\ \vdots & \vdots & \vdots & \vdots & \vdots \\ {f}_{SA}& {f}_{SB}& {f}_{SC}& \cdots & {f}_{ST}\\ {f}_{TA}& {f}_{TB}& {f}_{TC}& \cdots & {f}_{TT}\end{array}\right],$$

2 $${Z}_{xy}= \frac{0-(E({\psi }_{x})-E({\psi }_{y}))}{{\sigma }_{{\psi }_{x-y}}}= \frac{0-({\overline{\psi }}_{x}-{\overline{\psi }}_{y})}{{\sigma }_{{\psi }_{x-y}}},$$

3 $${\overline{\psi }}_{x}-{\overline{\psi }}_{y}={Z}_{xy} \sqrt{{\sigma }_{{\overline{\psi }}_{x}}^{2}+{\sigma }_{{\overline{\psi }}_{y}}^{2}-2{r}_{{\overline{\psi }}_{x}{\overline{\psi }}_{y}}{\cdot \sigma }_{{\overline{\psi }}_{x}}\cdot {\sigma }_{{\overline{\psi }}_{y}} ,}$$

4 $${Z}_{xy}= \frac{1}{\sqrt{2}}\left({\overline{\psi }}_{x}-{\overline{\psi }}_{y}\right),$$

5 $$\left[\begin{array}{ccccc}\frac{1}{\sqrt{2}}({\overline{\psi }}_{A}-{\overline{\psi }}_{A})& \frac{1}{\sqrt{2}}({\overline{\psi }}_{A}-{\overline{\psi }}_{B})& \frac{1}{\sqrt{2}}({\overline{\psi }}_{A}-{\overline{\psi }}_{C})& \cdots & \frac{1}{\sqrt{2}}({\overline{\psi }}_{A}-{\overline{\psi }}_{T})\\ \frac{1}{\sqrt{2}}({\overline{\psi }}_{B}-{\overline{\psi }}_{A})& \frac{1}{\sqrt{2}}({\overline{\psi }}_{B}-{\overline{\psi }}_{B})& \frac{1}{\sqrt{2}}({\overline{\psi }}_{B}-{\overline{\psi }}_{C})& \cdots & \frac{1}{\sqrt{2}}({\overline{\psi }}_{B}-{\overline{\psi }}_{A})\\ \vdots & \vdots & \vdots & \cdots & \vdots \\ \frac{1}{\sqrt{2}}({\overline{\psi }}_{S}-{\overline{\psi }}_{A})& \frac{1}{\sqrt{2}}({\overline{\psi }}_{S}-{\overline{\psi }}_{B})& \frac{1}{\sqrt{2}}({\overline{\psi }}_{S}-{\overline{\psi }}_{C})& \cdots & \frac{1}{\sqrt{2}}({\overline{\psi }}_{S}-{\overline{\psi }}_{T})\\ \frac{1}{\sqrt{2}}({\overline{\psi }}_{T}-{\overline{\psi }}_{A})& \frac{1}{\sqrt{2}}({\overline{\psi }}_{T}-{\overline{\psi }}_{B})& \frac{1}{\sqrt{2}}({\overline{\psi }}_{T}-{\overline{\psi }}_{C})& \cdots & \frac{1}{\sqrt{2}}({\overline{\psi }}_{T}-{\overline{\psi }}_{T})\end{array}\right]\cdot \left[\begin{array}{c}1\\ 1\\ \vdots \\ 1\\ 1\end{array}\right]= \left[\begin{array}{c}\frac{1}{\sqrt{2}}(n{\overline{\psi }}_{A}-\sum_{i=A}^{T}{\overline{\psi }}_{i})\\ \frac{1}{\sqrt{2}}(n{\overline{\psi }}_{B}-\sum_{i=A}^{T}{\overline{\psi }}_{i})\\ \vdots \\ \frac{1}{\sqrt{2}}(n{\overline{\psi }}_{S}-\sum_{i=A}^{T}{\overline{\psi }}_{i})\\ \frac{1}{\sqrt{2}}(n{\overline{\psi }}_{T}-\sum_{i=A}^{T}{\overline{\psi }}_{i})\end{array}\right]= \left[\begin{array}{c}{\overline{\psi }}_{A}-c\\ {\overline{\psi }}_{B}-c\\ \vdots \\ {\overline{\psi }}_{S}-c\\ {\overline{\psi }}_{T}-c\end{array}\right].$$

The $\overline{\psi }$ derived in Eq. (5) is the psychological distance. The mean of the 10th (${\overline{\psi }}_{J}$) and 11th (${\overline{\psi }}_{K}$) pictures on the psychological distance map is the desired median of psychological distance. This median served as the dividing line between higher and lower positive emotion pictures. Each participant had their own psychological distance map, and thus it is possible that a particular picture was higher than the median for one participant but lower than the median for another participant.

#### Quantifying type-I performance and meta-emotion

After acquiring emotion and confidence judgment data, we quantified participants’ meta-level performance based on SDT^5^. For any trial, if picture-induced emotion was higher than the median based on the participant’s own psychological distance map, and the participant also judged induced emotion to be higher (lower) than their personal median, this trial would be counted as a hit (miss). On the other hand, if picture-induced emotion was lower than the median based on the participant’s own psychological distance map and the participant’s response was lower (higher), this trial would be counted as a correct rejection (false alarm). The difference between the *z*-transformed hit and false alarm rates is the dʹ, which thus serves as the behavioral index of the type-I emotion task.

Each participant’s metacognitive efficiency was calculated using SDT and hierarchical Bayesian estimation (through the HMeta-d toolbox)^3^. The use of hierarchical Bayesian estimation to derive metacognitive efficiency offers several advantages, including (1) the ability to obtain more accurate parameters when the number of trials per participant is limited, (2) the incorporation of uncertainty in group-level parameter estimation, and (3) the avoidance of edge correction and data modification. According to Fleming^3^, HMeta-d provides relatively good control (compared to other metacognitive efficiency models) of the false positive rate when the trial number is greater than 50 per participant. Therefore, we utilized 60 trials per participant in the emotion evaluation task to ensure the parameters obtained were stable and precise.

Two confidence judgment distributions were generated: one for *correct* type-I responses (hit and correct rejection) and one for incorrect type-I responses (miss and false alarm). Metacognition is better when a participant rates higher confidence in correct type-I responses while rating lower confidence for incorrect type-I responses. The normalized distance between the two confidence judgment distributions (the distance between the two peaks of the distributions) represents the “absolute” metacognitive sensitivity (*meta-d*ʹ), which indicates how much information the confidence rating captures from the type-I task performance (the *d*ʹ). Because *d*ʹ and *meta-d*ʹ have the same unit, further comparison between them is possible. Specifically, the “relative” metacognitive sensitivity regressed out the contribution of the type-I performance from the absolute metacognitive sensitivity, which indicates how much information was used in the meta-level judgment compared to the type-I emotion task^6^. Two methods can be used to calculate metacognitive efficiency: *Meta-d*ʹ*/d*ʹ (*M-ratio*) and *meta-d*ʹ*–d*ʹ (*M-diff*), using division and subtraction to normalize the type-II performance, respectively. Here, we compared the *d*ʹ, meta-*d*ʹ, *M-ratio*, and *M-diff* indices between Test 1 and Test 2 and their correlations to examine the test–retest reliability of our measures of meta-emotion.

## Results

**Figure 2:**
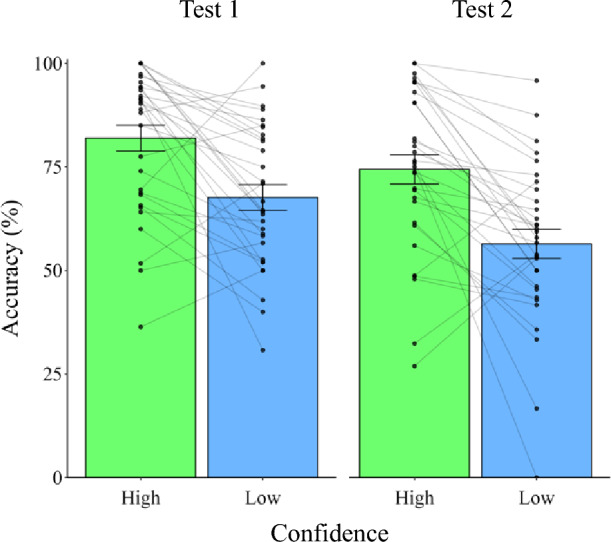
The accuracy in high-confidence and low-confidence trials in Test 1 and Test 2. Each dot indicates the performance of an individual. The error bars indicate one s.e.m.

To investigate whether individuals are capable of correctly identifying their own emotions, we examined participants’ meta-emotion ability using two methods. First, we compared between trials classified as high confidence (rated 3 or 4) and low confidence (rated 1 or 2) the accuracy of discrimination of whether affective pictures elicited higher or lower positive emotion than the median. If individuals possess meta-emotion ability, their accuracy should be higher in high-confidence trials than in low-confidence trials^28^. Results are shown in Fig. 2. The paired-*t*-test verified our hypothesis that discrimination accuracy is higher in the high-confidence than in the low-confidence trials in both Test 1 (*t*(31) = 9.29, *p* < 0.001, *d* = 1.64) and Test 2 (*t*(31) = 7.54, *p* < 0.001, *d* = 1.33). Second, we examined whether the meta-*d*ʹ was greater than zero to confirm that participants did not give their confidence rating by chance, with non-chance ratings indicating the existence of meta-emotion ability^29^. Accordingly, the meta-*d*ʹ was found to be greater than zero in both Test 1 (M = 1.3, SD = 0.97, *t*(31) = 7.57, *p* < 0.001, *d* = 1.34) and Test 2 (M = 1.06, SD = 1.16, *t*(31) = 5.14, *p* < 0.001, *d* = 0.91).

To reveal the stability and reliability of the task procedure, we conducted four paired-*t*-tests to compare the performance indices (i.e., *d*ʹ, meta-*d*ʹ, M-ratio, and M-diff) between Test 1 and Test 2 (Table 1). No significant differences in Test 1 and Test 2 were found in the meta-*d*ʹ (*t*(31) = 1.22, *p* = 0.231, *d* = 0.22), M-ratio (*t*(31) =  − 1.87, *p* = 0.071, *d* = 0.33), and M-diff (*t*(31) =  − 1.29, *p* = 0.205, *d* = 0.23), but a lower *d*ʹ was observed in Test 2, (*t*(31) = 2.66, *p* = 0.012, *d* = 0.47). Despite the lower* d*ʹ in Test 2, the metacognitive indices (meta-*d*ʹ, M-ratio, and M-diff) were similar across sessions and did not vary over time, suggesting that these measures were robust in quantifying individuals’ meta-emotion.

**Table 1:** Mean (and SD) of Test 1 and Test 2 in the performance indices.

	*d'*	meta-dʹ	M-ratio	M-diff
Test 1	1.75 (0.99)	1.29 (0.97)	0.72 (0.78)	− 0.46 (0.78)
Test 2	1.27 (1.08)	1.05 (1.16)	1.46 (2.22)	− 0.21 (1.06)

The mean (and SD) for M-ratio in Test 1 and Test 2 are 0.72 (0.81) and 1.04 (1.19) after removing the two outliers (see below).

**Figure 3:**
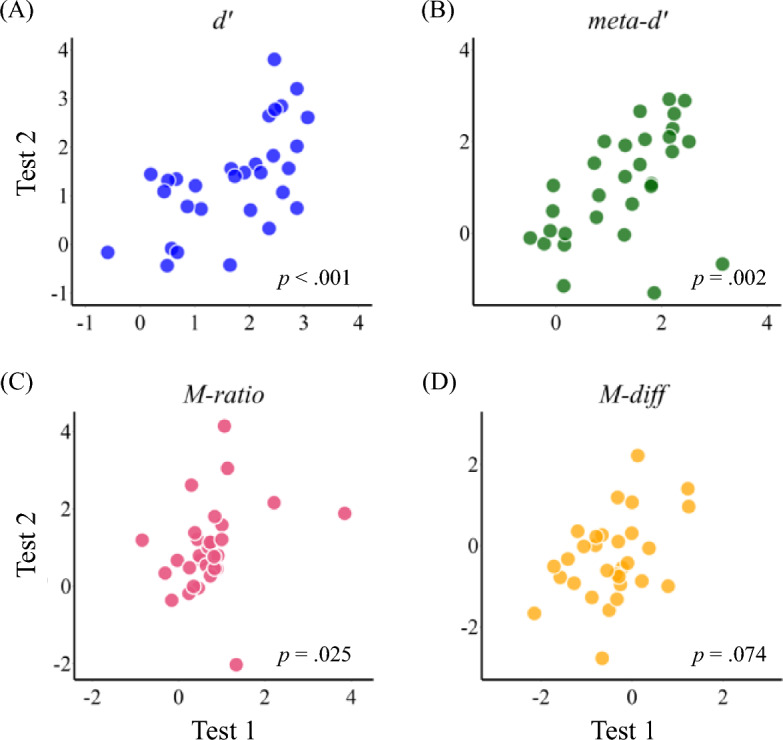
Test 1 and Test 2 results for (**A**) *d*ʹ, (**B**) meta-*d*ʹ, (**C**) M-ratio, and (**D**) M-diff. Test 1 data were mapped to the x-axis, while Test 2 data were mapped to the y-axis. Each dot represents individual performance. The *p*-values indicate the significance level derived from the Spearman correlation analysis.

Given the inherent characteristics of the modeling procedure in Hierarchical Bayesian estimation, where the same prior is used for parameter estimation across time points, it is important to note that the estimated indices may not be completely independent. To err on the side of caution, we conducted a non-parametric correlation analysis using Spearman correlation to examine the test–retest reliability across sessions (Fig. 3), following Fox et al.^30^ and Lund et al.^31^. After removing the two outliers (performance greater than 2.5 SD from the mean), we observed test–retest reliability in the indices: *d*ʹ (*ρ* = 0.61, *p* < 0.001), meta-*d*ʹ (*ρ* = 0.54, *p* = 0.002), M-ratio (*ρ* = 0.41, *p* = 0.025), and marginally with M-diff (*ρ* = 0.23, *p* = 0.074). We also validated our findings by using Pearson correlations as in Mazancieux et al.^6^—significant positive correlations between Test 1 and Test 2 were found for the *d*ʹ (*r* = 0.61, *p* < 0.001), meta-*d*ʹ (*r* = 0.49,* p* = 0.005), and M-diff (*r* = 0.41, *p* = 0.025), but not for M-ratio (*r* = 0.27, *p* = 0.145).

To ensure that the variance of the task difficulty in the calibration task across participants did not confound with the task performance in meta-emotion, we also looked at the relationship between the distribution of psychological distances (representing personal task difficulty) for each individual and their task performance. Specifically, we examined if a more distributed (large interquartile range, which could possibly be easier than the small interquartile range) psychological distance map led to better performance (due to greater separation among picture-induced emotions), thus leading to a positive correlation with the *d*ʹ of the task. We therefore used the interquartile range (IQR) as the index of the psychological distance distribution. The IQR of the psychological distance was defined as the difference between the Q3 and the Q1 of the psychological distance value (i.e., the 75% and 25% quartiles of the 20 values, respectively) within each individual. The IQR of the psychological distance (Test 1: M = 47.02, SD = 5.69, Test 2: M = 47.24, SD = 7.61) did not correlate with *d*ʹ (pretest: *r* = 0.23, *p* = 0.197, BF_10_ = 0.49; Test 2: *r* = 0.24, *p* = 0.176, BF_10_ = 0.53), meta-*d*ʹ (pretest: *r* = 0.24, *p* = 0.184, BF_10_ = 0.51; Test 2: *r* = 0.27, *p* = 0.132, BF_10_ = 0.65), M-ratio (pretest: *r* = 0.11, *p* = 0.548, BF_10_ = 0.26; Test 2: *r* = 0.03, *p* = 0.882, BF_10_ = 0.22), or M-diff (pretest: *r* < 0.01, *p* = 0.989, BF_10_ = 0.22; Test 2: *r* = 0.05, *p* = 0.793, BF_10_ = 0.23), where the Bayes Factor (BF_10_) was calculated using JASP^32^ with the default priors. The results suggest that the different distributions of the psychological distance across participants did not affect meta-emotion. Thus, the calibration task performance did not confound with the emotion evaluation task performance, and task difficulty (indexed by IQR) is a different concept from task performance (indexed by *d*ʹ).

Finally, we explored whether the questionnaires we administered and the task captured the same construct of meta-emotion. We examined the correlations between the scores for each questionnaire (Alexithymia scale, PANAS, and ERQ, see Table S1) and meta-emotion indices (*d*ʹ, meta-*d*ʹ, M-ratio, and M-diff) in both Test 1 and Test 2. In Test 1, there was a negative correlation between *d*ʹ and ERQ (*r* =  − 0.38, *p* = 0.034, BF_10_ = 1.90); in Test 2, there was a negative correlation between positive affectivity in the M-diff and PANAS (*r* =  − 0.42, *p* = 0.017, BF_10_ = 3.29). No other correlation reached statistical significance (all *ps* > 0.05, see Tables S2, S3). Since the two significant correlations were not reliable in Test 1 and Test 2 and only one of them was greater than 3 in BF_10_, we therefore consider the correlations here unstable and do not discuss them further.

## Discussion

The present study established a novel objective and quantitative measure of meta-emotion by conducting a meta-emotion experiment that included calibration and evaluation tasks. By constructing the psychological distance of emotional feelings toward pictures using the LCJ for each participant^18^, we captured each person’s sensitivity in identifying subjective feelings as indexed by *d*ʹ. Confidence ratings toward emotional judgments allowed us to quantify meta-emotion based on the SDT framework as indexed by *meta-d*ʹ. The metacognitive efficiency indices (i.e., M-ratio and M-diff) were computed accordingly, representing the normalized indices of how much information regarding one’s subjective feelings was used in the metacognitive judgment. We first confirmed that participants did possess meta-emotion ability, exhibited by higher accuracy in higher confidence trials and positive values (i.e., greater than 0) of *meta-d*ʹ. We also obtained robust estimations of the meta-*d*ʹ, M-ratio, and M-diff in Test 1 and Test 2. Through Spearman correlation analysis, positive correlations between Test 1 and Test 2 were observed in *d*ʹ, meta-*d*ʹ, M-ratio, and marginally with M-diff, indicating robust test–retest reliability for these measurements. No stable correlations between the scores of questionnaires and the indices of meta-emotion were observed across Test 1 and Test 2.

The test–retest reliability suggests that the measurements derived are stable and can be reproduced through the same experimental setup. In Fig. 3, when visually assessing the scatterplots, it appears that the distribution of M-ratio is more tightly clustered compared to other indices. However, some points exhibit noticeable deviations from the majority. Indeed, *M-ratio* can be inflated when the *d*ʹ is small, resulting in extreme values which may jeopardize the interpretation of *M-ratio*^2,33,34^. It is important to note that both M-ratio and M-diff are influenced by response bias, type-I performance, and number of trials^34,35^, while both of them have good precision in estimating the parameters; thus researchers should be attentive to their respective pros and cons when interpreting results.

### Quantifying the performance through the LCJ and SDT

In the present study, we captured the ability to monitor subjective emotional feelings through individual responses and LCJ. In addition, this is the first study to use an SDT-based approach to quantify emotional experience. Previous studies have used the coherence between physiological response and subjective rating as the index of emotion^36^; however, the coherence between physiological response and emotional ratings was sometimes not observed in another study using a similar approach^37^. Although interpretation of physiological signals can be a determining factor in emotional experience, the physiological response should not be considered as the only index of emotional experience^38,39^. In the current work, we avoid using an invasive approach to obtain the metacognition of emotion and rely simply on the task procedure to establish the ground truth of emotional feelings through LCJ, which is a breakthrough in the methodology used to measure metacognition of subjective feelings. Participants then compared their emotional experience to the median of the whole set and rated their confidence in this judgment. The type-II performance of subjective emotional feelings can thus be captured.

### Lack of correlations in meta-emotion with the questionnaires

We did not observe strong correlations between questionnaires and behavioral indices as expected. The inability of questionnaires to predict task performance has also been reported in interoception studies^16,40^. For example, Meessen et al.^41^ have suggested that the interoceptive *sensibility*, *accuracy*, and *awareness*—referring to the disposition, objective performance, and metacognitive judgment of interoception, respectively—indicate different aspects of interoceptive processes^41–43^. To be specific, interoceptive sensibility refers to the subjective measure of participants’ belief in their own interoceptive aptitude, irrespective of actual (objectively determined) interoceptive accuracy. Interoceptive awareness is defined as the metacognitive insight into one’s own interoceptive performance. Parallel to the emotion task here, the task we used likely captured the constructs of *emotion accuracy* (*d*ʹ) and *emotion awareness* (M-ratio and M-diff), while the questionnaires captured *emotion sensibility*. We did not consider this a lack of criterion-related validity; rather, such results imply that we should always pay attention to “what is measured” in the questionnaires and interpret the data accordingly. Moreover, self-reported questionnaires such as the Alexithymia scale may be susceptible to circular issues, as individuals may not report experiencing emotional issues if they are unable to identify these issues themselves in the first place. Future studies should be cautious when correlating questionnaire scores and task performance: the disposition of internal bodily sensations, detecting bodily sensations, and being aware or confident in perceiving these internal bodily signals represent different processes and should not be conflated^44^.

### The higher-order representation of emotional experience

In addition to the methodological advancement in the field of emotion and meta-emotion, our study also contributes to the understanding of emotional consciousness from a clinical and philosophical view. First, patients suffering from affective disorders may not convey or introspect their own emotions precisely, given that their monitoring system toward emotions is plausibly impaired. Hence, treatments aiming at enhancing metacognitive abilities should be emphasized rather than merely managing the physiological response resulting from external stimuli or internal thoughts^45^. Second, according to the higher-order theory of emotion consciousness^46^, emotional experiences are higher-order representations (HORs) instantiated in the cortical circuits. To be aware of this HOR (the emotional experience in this case), an HOR on top of the existing HOR is required (i.e., the meta-HOR). Despite the theory proposed, no experimental designs have been developed to capture this concept regarding meta-HOR. The current study demonstrated an empirical approach to quantify meta-emotion, which can also be used to quantify meta-HOR, to provide scientific evidence supporting the higher-order theory of emotional consciousness. Future studies can apply a similar design to verify the neural substrate of meta-emotion, which is considered the same hierarchical structure as that of metacognition of exteroception^46^.

### Future directions

The current study used LCJ and SDT to quantify meta-emotion with test–retest reliability. We focused mainly on the intensity of positive emotions, while other dimensions (e.g., arousal and dominance) and categories (e.g., negative) of emotion in the design were excluded. Indeed, in real life, we usually evaluate our performance over more than two categories of responses and situations^47^. We experience complex emotions daily, such as jealousy and guilt, among others. Future studies could include different domains of emotion in the experiment to provide a more comprehensive view of meta-emotion. Additionally, a limitation in our study lies in assuming that the psychological distance induced by the pictures remained constant throughout the single session. A more rigorous approach would involve re-measuring the psychological distance after completing the entire task to ensure that the midpoint of the psychological distance did not change during the task.

While the current design strives to capture the construct of meta-emotion, it is important to acknowledge that the measurement used here can be accounted for by other cognitive processes. For instance, participants may have been engaging in additional processes related to the emotional stimuli that are not directly linked to metacognition, or they might have employed specific strategies when providing their responses. Consequently, interpreting the results should be approached with caution, considering potential influences from these factors. Future studies can use the same stimuli with a different task instruction (e.g., rating the visual features of the pictures) and compare whether the metacognitive indices are different from the emotional ones.

The methods combining the LCJ and SDT here can be generalized to measure the metacognition of other subjective feelings, opening a new window in studying the metacognition of subjective experiences. For example, when it comes to aesthetics, individuals have their own subjective criteria for the evaluation of artwork^48,49^. Metacognition of aesthetics can thus be measured through the same procedure. Monitoring and knowing our subjective feelings (such as emotion, preference, and aesthetics) is critical since such feelings are highly involved in the reward circuits^50^. By identifying what genuinely interests us, we can reinforce the positive feedback from the external world or make the right decision, increasing happiness and welfare in our daily lives.

### Supplementary Information

Supplementary Tables.

## Data Availability

The data and code for this study are accessible at https://osf.io/hp4e2/?view_only=e7b0b7c18a9f4a00b21b2b53f5711b91. The materials are not publicly accessible as the copyright belongs to a third-party research group.
