# Supplementary material for: Exploring quantitative measures in metacognition of emotion
Source: Sci Rep. 2024 Jan 23;14:1990. doi: 10.1038/s41598-023-49709-7 (PMC10805884; doi:10.1038/s41598-023-49709-7)
Supplement: Supplementary file 1 — Supplementary Tables. [file 41598_2023_49709_MOESM1_ESM.pdf]

Appendix

Table S1.

The questionnaire scores in Test 1 and Test 2

	Test 1 mean	Test 2 mean	*t*-value	*p*-value
Alexithymia scale	51.15	51.42	0.20	0.85
PANAS-positive	24.70	25.12	0.33	0.75
PANAS-negative	19.61	18.84	-0.48	0.64
ERQ	41.55	41.64	0.09	0.93

Table S2.

The correlation coefficients between the questionnaire scores and the task performance in Test 1

	Test 1			
	Alexithymia	PANAS-positive	PANAS-negative	ERS
d'	0.14 (0.30)	-0.01(0.22)	0.10 (0.26)	-0.38 (1.90)
meta-*d'*	0.02 (0.22)	-0.02 (0.22)	0.10 (0.25)	-0.16 (0.32)
M-ratio	-0.04 (0.22)	-0.13 (0.28)	-0.18 (0.36)	0.17 (0.34)
M-diff	-0.16 (0.32)	-0.02 (0.22)	-0.25 (0.56)	0.28 (0.67)

Note. The Bayes Factor (BF_{10}) is provided in the paratheses.

Table S3.

The correlation coefficients between the questionnaire scores and the task performance in Test 2

	Test 2			
	Alexithymia	PANAS-positive	PANAS-negative	ERS
d'	0.22 (0.43)	0.06 (0.23)	0.09 (0.25)	-0.13 (0.28)
meta-*d'*	0.07 (0.24)	-0.33(1.08)	0.03 (0.22)	-0.05 (0.23)
M-ratio	-0.09 (0.25)	-0.27 (0.64)	0.19 (0.37)	0.12 (0.27)
M-diff	-0.14 (0.30)	-0.42 (3.29)*	-0.06 (0.23)	0.07 (0.24)

Note. The Bayes Factor (BF_{10}) is provided in the paratheses. * indicates $p < .05$ in the frequentist statistics and $BF_{10} > 3$ in the Bayesian statistics.
